# Supplementary material for: Splice-Junction-Based Mapping of Alternative Isoforms in the Human Proteome
Source: Cell Rep. Author manuscript; Available in PMC 2020 Jan 15. (PMC6961840; doi:10.1016/j.celrep.2019.11.026)
Supplement: 2 [file NIHMS1546469-supplement-2.zip › DF1/PXD010154/Esophagus/Esophagus_2_STK39_VKEENPEGPPNANEDYR.pdf]

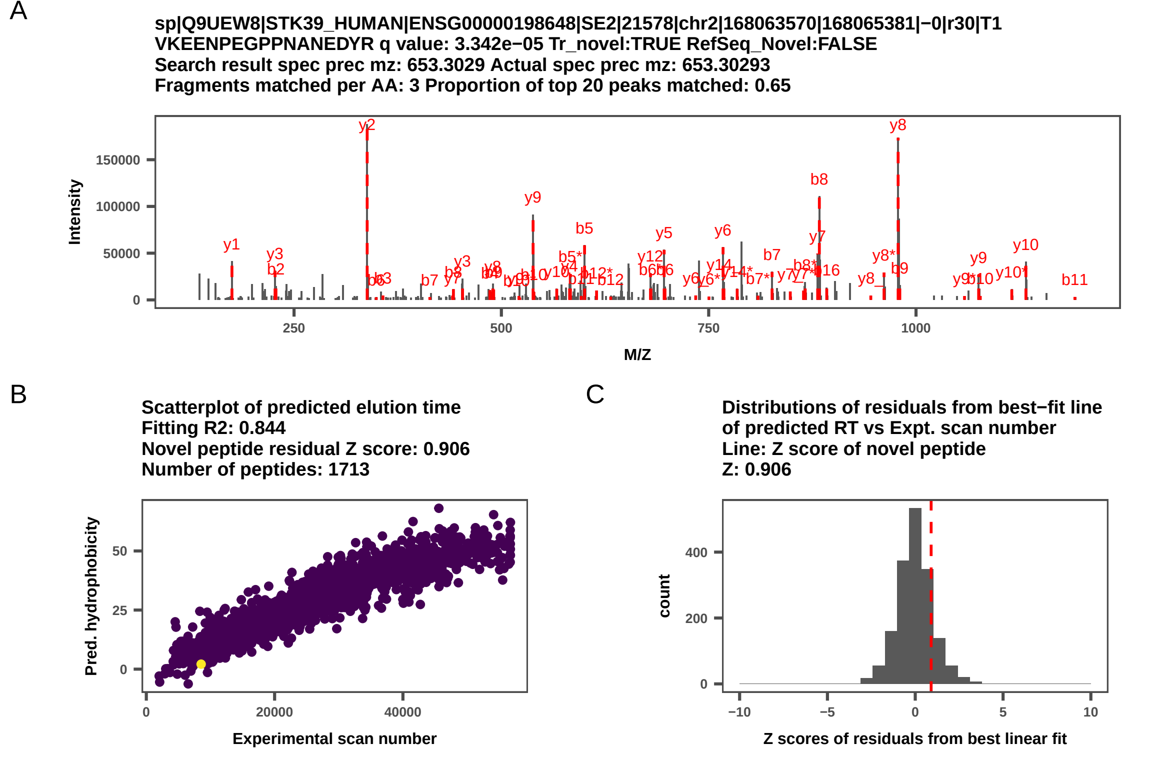
A
sp|Q9UEW8|STK39_HUMAN|ENSG00000198648|SE2|21578|chr2|168063570|168065381|−0|r30|T1
VKEENPEGPPNANEDYR q value: 3.342e−05 Tr_novel:TRUE RefSeq_Novel:FALSE
Search result spec prec mz: 653.3029 Actual spec prec mz: 653.30293
Fragments matched per AA: 3 Proportion of top 20 peaks matched: 0.65
Intensity
150000
100000
50000
0
250
500
750
1000
M/Z
y2
y8
b8
y9
b5
y5
y6
y7
y1
y3
b2
b7
y10
y9
b11
B
Scatterplot of predicted elution time
Fitting R2: 0.844
Novel peptide residual Z score: 0.906
Number of peptides: 1713
Pred. hydrophobicity
50
25
0
0
20000
40000
Experimental scan number
C
Distributions of residuals from best−fit line
of predicted RT vs Expt. scan number
Line: Z score of novel peptide
Z: 0.906
count
400
200
0
−10
−5
0
5
10
Z scores of residuals from best linear fit
